# Antibiotic and Heavy Metal Co-Resistant Strain Isolated from Enrichment Culture of Marine Sediments, with Potential for Environmental Bioremediation Applications

**DOI:** 10.3390/antibiotics12091379

**Published:** 2023-08-29

**Authors:** Han-Sheng Zhu, Xiao Liang, Jun-Cheng Liu, Han-Yang Zhong, Yuan-Hang Yang, Wen-Peng Guan, Zong-Jun Du, Meng-Qi Ye

**Affiliations:** 1 Marine College, Shandong University, Weihai 264209, China; zhuhansheng_0405@163.com (H.-S.Z.);; 2 SDU-ANU Joint Science College, Shandong University, Weihai 264209, China; 3 Weihai Research Institute of Industrial Technology, Shandong University, Weihai 264209, China; 4 Shenzhen Research Institute, Shandong University, Shenzhen 518057, China

**Keywords:** bioremediation, multidrug resistance, antibiotics, heavy metals, *Rossellomorea*

## Abstract

Antibiotics and heavy metals have caused serious contamination of the environment and even resulted in public health concerns. It has therefore become even more urgent to adopt a sustainable approach to combating these polluted environments. In this paper, we investigated the microbial community of marine sediment samples after 255 days of enrichment culture under Cu (II) and lincomycin stress and ZC255 was the most resistant strain obtained. The 16S rRNA gene sequence confirmed that it belonged to the genus *Rossellomorea*. Strain ZC255 was resistant to 12 kinds of antibiotics, and had a superior tolerance to Cu (II), Pb (II), Ni (II), Zn (II), Cr (III), and Cd (II). Moreover, it exhibits strong bioremoval ability of Cu and lincomycin. The removal efficiency of Cu (II) and lincomycin can achieve 651 mg/g biomass and 32.5 mg/g biomass, respectively. Strain ZC255 was a promising isolate for pollution bioremediation applications.

## 1. Introduction

For the past half-century, the rapid development of agriculture, culture fishery, and industry, especially in coastal areas, has led to increasingly serious pollution of the marine environment with heavy metals worldwide [1]. Metals are essential to the biological functioning of plants and animals, but if levels are too high, they can interfere with the metabolic reactions of biological systems [2]. Marine species diversity and ecosystems can be damaged by toxic heavy metals [3] such as Pb, Ni, and Cu, which can reduce species diversity and abundance, leading to the degradation of marine ecosystems. In addition, the metals can accumulate in humans and animals through food chains [4], leading to potential health risks and harmful effects on humans and animals [5].

At the same time, the misuse or overuse of antibiotics in the mariculture industry for the prevention and treatment of cultured diseases is a growing problem [6], and large amounts of antibiotics remain in the marine ecosystem due to the connectivity between the aquaculture area and the marine environment [7]. They may inhibit the activity of beneficial microorganisms in the marine, leading to the deterioration of the microecological environment and interfering with or even blocking the circulation of ecosystem materials and energy flow [8]. Antibiotics tend to be more enriched in the sediment [9] because the bottom sediments are less mobile. Antibiotics in the sediments are difficult to diffuse and migrate, and excessive antibiotic accumulation will have a huge impact on the benthic environment and the microbial community. Antibiotics are introduced into the marine environment in different ways. Accumulation in sediments can have long-term adverse effects on aquatic life [10], it causes serious pollution of the marine environment and also has a major impact on human health when ingested by humans [11]. Overall, the antibiotic resistance pandemic is an extremely radical and complex crisis for human and environmental health [12].

Related studies have shown that heavy metals can combine with antibiotics in compound pollution [13], synergistic selection pressure between heavy metals and antibiotics increases the population of co-resistant microorganisms in the environment, impairs microbial communities and the expression of genes that function in the nitrogen cycle, and affects microorganisms in the marine environment, causing changes in the structure and diversity of natural microbial communities and affecting the ecological environment [14].

Several reports indicate that the recurrent detection of antibiotic residues is frequently observed in aquatic environments, including freshwater and coastal areas [15,16]. Among the antibiotics, lincomycin (8.55 ng/L) was detected as the main compound in the seawater of Jiaozhou Bay [17]. Lincomycin is a naturally sourced lincosamide antibiotic. Its inhibitory effect is derived from blocking protein synthesis, and its antibacterial effect against Gram-positive bacteria is similar to erythromycin [18]. In addition, it is persistent in the environment [19]. Lin et al. detected high levels of lincomycin residues (111.667 μg/L) in aquaculture areas and rivers [4]. It had been reported to have a high ecological risk to relevant algae [20]. These indicate that seafood consumption poses considerable risks to human health. Heavy metals are present in soluble form and exhibit extreme toxicity to microbial life activities. Cu is an indispensable element in many biochemical processes. With the aggravation of heavy metal pollution in the marine environment, Cu concentration shows a positive trend, which has a significant impact on the physiological activities of marine organisms [21,22].

Antibiotics and heavy metal pollution have attracted considerable attention worldwide [23]. Antibiotics pose a high risk to microalgae and aquatic animals, as shown by Li et al. in a review of risk assessments of water bodies worldwide [24]. A review by Rakib et al. [25] found that ecosystems along the coast of Bangladesh are heavily contaminated due to the high accumulation of metals, with associated threats to public and ecological health. Nowadays, conventional methods (chemical method, ion exchange, and membrane filtration) show low efficiency in removing antibiotics and heavy metals. The chemical method is a simple and low-cost process, but it can only treat industrial wastewater that is highly concentrated, simple in content, and may produce hazardous substances. Ion exchange has a high recovery rate, but it cannot treat highly concentrated solutions and the substrate is easily contaminated with organics and other solids in the wastewater [26]. Membrane filtration technology is efficient and easy to operate, but requires high maintenance and operating costs [27]. Bioremediation, by using the degrading ability and adsorption or accumulation ability of microorganisms, is an efficient, cost-effective, and more sustainable alternative to treat contaminated media. Bacteria can adsorb, accumulate (through processes independent of passive metabolism or dependent on active metabolism), and transform most heavy metals and antibiotics into harmless forms. Heavy metals and antibiotic-resistant bacteria have shown very efficient and various mechanisms in tolerating high concentrations of toxic metals and antibiotics [28]. Therefore, they have important potential for bioremediation. The resistance and accumulation capacity of the bacteria ensures that they can be used for the bioremediation of contaminated environments. Antibiotic resistance genes in bacteria arise from the selective pressure or horizontal gene transfer (HGT) of antibiotics [29], and heavy metals can enhance selection for antibiotic resistance in the environment [30]. HGT carries the risk of spreading resistance [31], which needs to be taken into account.

In this study, we collected marine sediments from the coast of Xiao Shi Island, Weihai, China and obtained a multidrug-resistant strain by isolation after 255 days of enrichment culture in an enrichment medium rich in Cu (II) and lincomycin. Relevant physiological, biochemical, and molecular biological studies were conducted to evaluate the potential of this strain for bioremediation. Our study will provide excellent strains for the potential application in bioremediation of heavy metals and antibiotics, which is of great significance in the field of bioresource environmental protection.

## 2. Results and Discussion

### 2.1. Analysis of the Community Structure of the Isolated Strains

**Figure 1:**
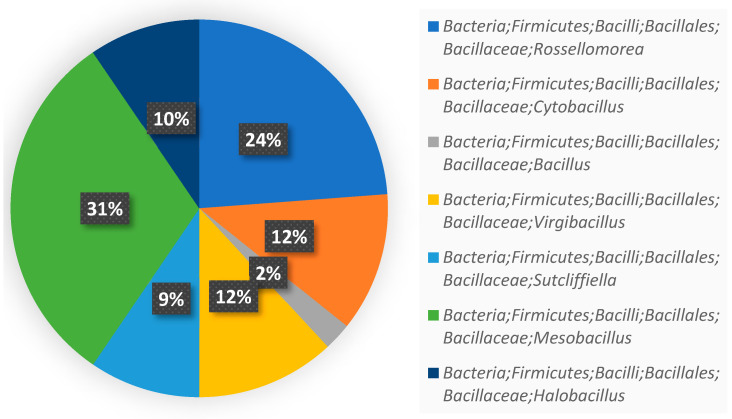
Composition of the screened strains at the genus level.

The bacteria in the enriched culture were isolated using Marine agar 2216 (MA, Becton-Dickinson, Franklin Lakes, NJ, USA) which contained CuSO_4_ (400 mg/L) and lincomycin (100 mg/L). The composition of the obtained bacteria was analyzed by sequencing results of the 16S rRNA gene (Figure 1).

The separation screening yielded a total of 96 strains with good resistance. They all belong to Phylum *Firmicutes* Class *Bacilli* Order *Bacillales* Family *Bacillaceae*, which indicates that the *Bacillaceae* family group has good resistance to both lincomycin and Cu (II), and the top two abundant genera were *Mesobacillus* (34%) and *Rossellomorea* (25%). The isolates obtained under polymetallic conditions by Seralathan et al. [32] were all *Bacillus*, which was similar to our study. Moreover, Chari et al. [33] isolated 46 marine heterotrophic bacteria with antibiotic resistance from the Palk Bay sediments, with the majority of the resistant strains belonging to *Bacillales* (74%). These studies showed that the *Bacillus* species found in the marine environment have a good level of resistance activity. This also suggests that the addition of lincomycin and Cu (II) to the enrichment culture may have a relatively large impact on microbial community composition.

Among 96 strains, the fastest growing orange–red spherical single colony was obtained during the screening process and named ZC255. At the same time, the strain was more tolerant to temperature and salinity than other isolated strains under different salinity and temperature conditions. During further screening, when the concentration of CuSO_4_ was increased to 500 mg/L and the concentration of lincomycin reached 300 mg/L, only 10 strains survived but grew slowly except for strain ZC255. It can be found that after 255 days of enrichment culture, the resistance of strain ZC255 was much greater than other strains. Thus, it can be speculated that there is no mobile genetic element in the DNA of strain ZC255.

### 2.2. Biochemical and Phenotypic Properties of Strain ZC255

In order to better understand the isolate for future applications, a series of physiological and biochemical properties of the isolate were studied. Strain ZC255 is a Gram-positive aerobic rod-shaped bacterium (Figure S1). Colonies on MA were raised, smooth, orange–red, circular with entire margins, and 1–2 mm in diameter after 48 h growth at 30 °C. It was grown at aerobic conditions ranging from 15 °C to 45 °C with an optimum temperature of 37 °C. The strain reached intensive growth after 48 h of incubation on MA medium at 37 °C. In terms of NaCl tolerance, strain ZC255 was able to tolerate up to 18% NaCl (*w*/*v*) and was able to grow in absence of NaCl. Its optimal NaCl concentration is 3% NaCl (*w*/*v*). Growth was observed at pH from 6.0 to 9.0 (optimum pH at 7.0–8.0) (Figure S2). Oxidase activity, hydrolysis of Tween 60, caseins, starch, CM-cellulose, and alginate were positive, but catalase activity, nitrate reduction, and hydrolysis of Tween 20, 40, and 80 were negative.

The biochemical characteristics of the strain were tested using the API reagent Strips and Biolog GEN III MicroPlate. Using the API 20E, citrate utilization, urease, tryptophan deaminase, and gelatin were positive for ZC255. Using the Biolog GEN III MicroPlate, d-maltose, d-trehalose, d-cellobiose, sucrose, d-turanose, stachyose, positive control, N-acetyl-d-glucosamine, N-acetyl-*β*-d-mannosamine, N-acetyl-d-galactosamine, N-acetyl neuraminic acid, 1% NaCl, d-mannose, d-fructose, d-galactose, 3-methyl glucose, d-fucose, L-fucose, L-rhamnose, inosine, d-sorbitol, myo-inositol, pectin, d-gluconic acid, acetoacetic acid, and acetic acid tests were positive. Using the API ZYM, 2-Naphthyl octanoate, 2-Naphthyl tetradecanoate, and N-Naphthyl-phosphate were positive. Using the API 50CH, glycol, d-ribose, d-galactose, d-glucose, d-fructose, d-mannose, methyl-*α*d-glucopyranoside, N-acety lglucosamine, amygdalin, ARBULIN, heptachy botrysum and iron citrate, salicin, d-cellobiose, d-maltose, d-lactose, d-midiose, d-sucrose, d-alginate, d-sonotriose, d-cottonose, starch, glycogen, d-gentian disaccharide, d-turanose, d-tagatose, and 5 Potassium ketogluconate were positive (Table S1). 

Compared with other species [34,35,36,37] in this genus, strain ZC255 is more tolerant to temperature, salinity, and pH, and its enzyme-producing ability is outstanding; it can also utilize a variety of substrates for growth, demonstrating its strong growth ability. These confirm that strain ZC255 has good tolerance properties and can survive widespread in nature. Among them, the physiological and biochemical properties of *Rossellomorea arthrocnemi* [37], which was used as a phytoremediation tool in heavy metal-contaminated soil, were very similar to strain ZC255. Strain ZC255 has a strong potential application value, especially for bioremediation in terms of antibiotic and heavy metal contamination.

### 2.3. Phylogenetic Analysis of the Strain

**Figure 2:**
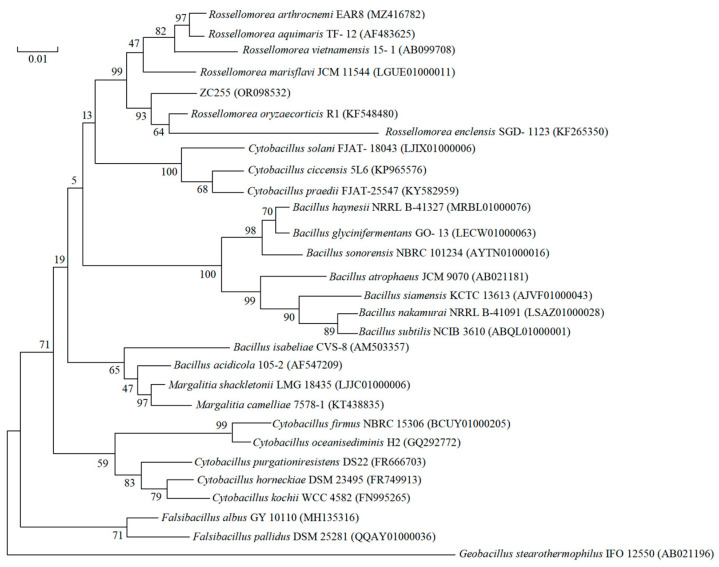
Phylogenetic tree based on 16S rRNA gene sequence of ZC255. (Phylogenetic tree constructed with 16S rRNA gene sequences using the neighbor-joining method showed the position of strain ZC255 among related taxa. Gen-Bank accession numbers of 16S rRNA gene sequences are given in parentheses. Bar, 0.01 substitutions per nucleotide position.)

The 16S rRNA gene sequence of strain ZC255 was amplified by PCR and was sequenced to be 1521 bp in length. In addition, a partial sequence of the bacteria has been deposited in the GenBank public database under accession number OR098532. The phylogenetic tree (Figure 2) was constructed by searching and matching the identity sequence by EZbioCloud. The 16S rRNA gene sequence confirmed that it belonged to the Phylum *Firmicutes* Class *Bacilli* Order *Bacillales* Family *Bacillaceae* genus *Rossellomorea*, showing the highest sequence similarity to *Rossellomorea oryzaecorticis* (99.46%) [38]. The species of Genus *Rossellomorea* were initially classified in genus *Bacillus.* In 2020, they were proposed as novel *Bacillaceae* genus *Rossellomorea* [39]. The most distinguishing feature of most members of the family *Bacillaceae* (phylum *Firmicutes*) is the ability to form heat-, radiation-, chemical-, and drought-resistant endospores, enabling them to survive for long periods under adverse conditions. This confirms that strain ZC255 has a high resistance to stress, which could explain why it has better co-resistance to heavy metals and antibiotics.

### 2.4. Biofilm Formation Assay

**Figure 3:**
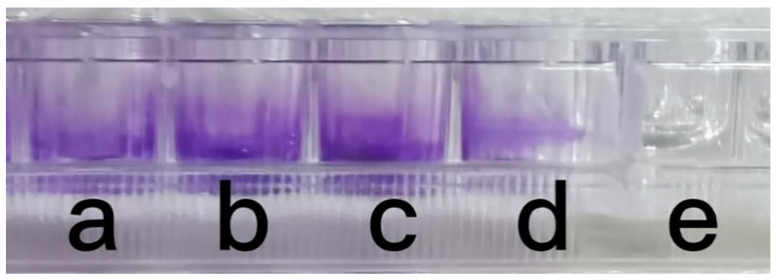
Biofilm formation test of strain ZC255 ((**a**–**d**) Cu (II), (**e**) control).

As shown in Figure 3, strain ZC255 has the ability to form biofilms in the presence of heavy metals. The results showed that no biofilm was formed in the absence of heavy metals, while biofilm was formed in the presence of Cu (II). Bacteria tend to form biofilms under different pressures that include the presence of toxic substances such as antibiotics and heavy metals, as well as oxygen limitation [40]. *Bacillaceae* strains had been reported to resist stress from heavy metals by forming biofilms [41]. The biofilm formation is a defense mechanism for bacteria, which can increase the survival rate of these bacteria under stress [42]. Thus, these confirmed that the biofilm formation by strain ZC255 in the pharmaceutical environment will play an important role in bioremediation.

### 2.5. Drug Resistance of Strain ZC255

It is important to assess the applicability of strains in bioremediation by understanding their ability to grow in the presence of contaminants. The experimental data (Table 1) showed that strain ZC255 was resistant to multiple heavy metals. The order of metal tolerance was as follows: Cu (II) > Zn (II) > Ni (II) > Pb (II) > Cr (III) > Cd (II) > Mn (II). The high tolerance to Cu (II), with an MIC of 1600 mg/L, may have been due to the accommodation of high levels of Cu contamination in the marine sediment. According to some reported strains, He et al. [43] found the MIC of Cu (II) for the growth of *Geotrichum* sp. strain CS-67 to be 350 mg/L and the MIC of Ni (II) for the growth of *Geotrichum* sp. strain CS-67 to be 100 mg/L; Ma et al. [44] found the MIC of Cd (II) for the growth of *Bacillus licheniformis* strain PB3 to be 50 mg/L. Through comparison with these strains, strain ZC255 had a superior tolerance to Cu (II), Cd (II), Pb (II), Cr (III), Zn (II), and Ni (II), which means this strain has great potential for the bioremediation of Cu contamination. There is a paucity of literature on Marine sediment isolates being tolerant to heavy metals [33]. It is well known that marine microorganisms are constantly exposed to different heavy metals. They are also famous as their various metal detoxification mechanisms [45]. Thus, they acquire resistance to heavy metals through this mechanism.

**Figure 4:**
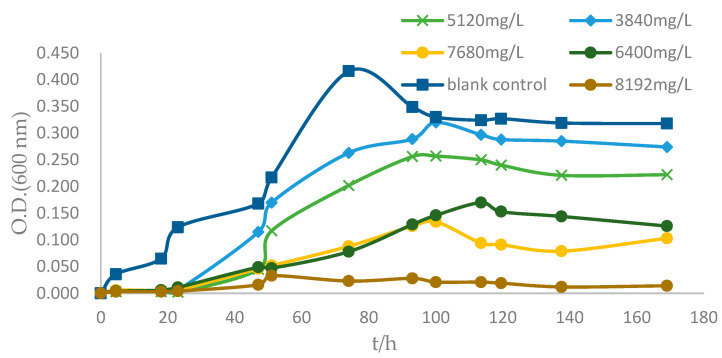
Effect of different concentrations of Cu (II) on the growth of strain ZC255.

When the Cu (II) concentration was 0, strain ZC255 began to enter the logarithmic growth phase at about 10 h, the growth rate slowed down at about 70 h, and gradually entered the stable phase. When the Cu (II) concentration was 3840–7680 mg/L, there was little effect on the ZC255 logarithmic phase and the stable phase; when the Cu (II) concentration reached 8192 mg/L, the growth of ZC255 was not obvious (Figure 4). Overall, the growth of the bacterial strain was suppressed by Cu (II), and the degree of inhibition gradually increased with the increase in Cu (II) concentration. However, strain ZC255 can still undergo normal growth and reproduction after a certain period of adjustment and then enter a stable period, indicating that it still has some resistance to Cu (II). Strain ZC255 has the MTC of 7680 mg/L for Cu (II).

The reference standards for the antimicrobial range of the disk method for drug susceptibility testing of resistant bacteria are shown in Table S2.

Combined with the presentation data, the strain ZC255 showed resistance to lincomycin, norfloxacin, kanamycin, ofloxacin, polymyxin B, ceftriaxone, erythromycin, streptomycin, neomycin, tobramycin, tetracycline, and gentamycin, but showed sensitivity to carbenicillin, vancomycin, ampicillin, penicillin, chloramphenicol, and clarithromycin (Table 2 and Figure S3). This indicated that strain ZC255 was multidrug resistant.

The MIC of lincomycin was further determined to be 100 mg/L. Salvatore et al. [46] evaluated the MIC of 154 isolates of *Mycoplasma pneumoniae* collected from Italy from 2012–2017, 87% of which had MIC values ≤0.5~1 mg/mL for lincomycin, but 7 strains (4.5%) had MIC values of 32 mg/mL for lincomycin. In comparative analysis with these strains, strain ZC255 showed a high resistance to lincomycin. Monitoring of antibiotic resistance in two recreational waters in Louisiana found that both biofilm isolates and water isolates had low resistance to some antibiotics, meaning that most isolates were sensitive to the antibiotic and successfully killed the bacteria [47]. This indicates that it is not easy to isolate a strain that is resistant to multiple antibiotics, and therefore strain ZC255 is particularly important in the management of antibiotic-contaminated environments. 

**Figure 5:**
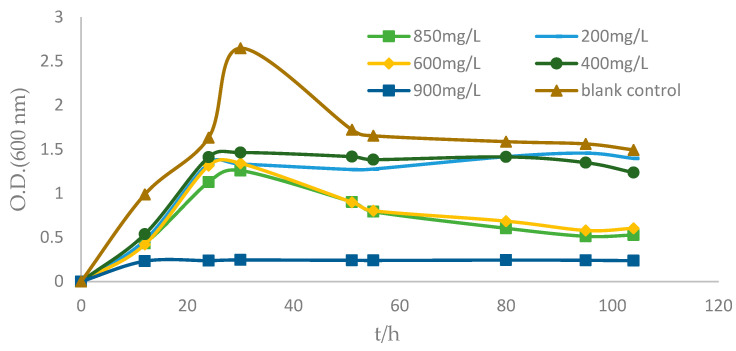
Effect of different concentrations of lincomycin on the growth of strain ZC255.

When the lincomycin concentration was 0–850 mg/L, there was little effect on the ZC255 logarithmic phase and the stable phase; when the lincomycin concentration reached 900 mg/L, the ZC255 phase was not obvious (Figure 5). For the MTC of lincomycin, the strain ZC255 was 850 mg/L.

Strain ZC255 remained stable growth after 100 h of growth in 1/10 MB; however, no signs of strain growth were observed in the control cultures. According to the change in the OD_600_ value in the concentration gradient of each experimental group at the same period, strain ZC255 can greatly resist lincomycin. Limited information has been known about lincomycin-degrading microorganisms in recent years; the strains that can bioremove lincomycin are in deficiency. Strain ZC255 has the potential to remove lincomycin and perform the bioremediation of marine environments contaminated by lincomycin. 

### 2.6. Evaluation of the Bioremoval Capacity of Strain ZC255

The bioremoval efficiency of Cu and lincomycin by strain ZC255 is shown in Table 3 and Table 4. According to the experimental data, the removal rate and removal capacity amounts of different concentrations of Cu (II) and lincomycin were different. When the initial concentration was 1200 mg/L, the bioremoval capacity of Cu (II) by strain ZC255 reached 350 ± 2.6 mg/g biomass, with a bioremoval rate of 16.67%; when the initial concentration was 3420 mg/L, the bioremoval capacity reached 651 ± 1.7 mg/g biomass, with a bioremoval rate of 5.43%. The absolute adsorption of lincomycin reached 32.5 ± 0.4 mg/g biomass at an initial concentration of 424 mg/L, with a bioremoval rate of 9.19%, while the absolute adsorption reached 8.1 ± 0.1 mg/g biomass at an initial concentration of 199 mg/L, with a bioremoval rate of 6.03%. It can be found that with the increase in drug concentration, although the removal rate decreased due to the inhibitory effect of the drug on the bacterial strain, the biological removal ability of the bacterial strain showed a significant enhancement. Statistical analysis showed a significant difference between initial and post-treatment dosages (*p* < 0.001). 

Iskandar et al. [48] isolated a filamentous fungi from freshwater ecosystem. The metal bioremoval of the fungi which occurred at 200 mg/L Cu (II) was (20.910 ± 0.581) mg/g. Wong et al. [49] found that fungal isolates *Xylaria* sp. NA40 had a high bioremoval capacity (73.26 ± 1.61 mg Cu per g biomass) for live biomass. Wang et al. [50] isolated the *Clostridium* sp. strain LCM-B from lincomycin mycelial residue (LMR), which could remove about 15.61 ± 3.54% at the initial concentration of 500 mg/L of lincomycin after incubation for 10 d, and its bioremoval capacity reached 10 mg/g. In this study, the comparison of the bioremoval capacity shows that the strain ZC255 had more outstanding bioremoval capacity for copper and lincomycin. The bioremoval capacity of the strain in this study was high enough to be used for practical applications. According to recent studies [51,52], strain ZC255 has considerable potential for the remediation of industrial, marine, and domestic wastewater due to its physiological and biochemical properties and its ability to bioremove Cu (II) and lincomycin. These results indicate that strain ZC255 has strong potential for bioremediation in heavy metal- and antibiotic-contaminated environments.

## 3. Materials and Methods

### 3.1. Sampling and Enrichment

The sediment samples were collected from the coast of Xiao Shi Island, Weihai, China (37°52′68″ N, 122°01′10″ E), at a depth of about 5–10 cm. About 20 g of sediment samples was placed in sterilized 250 mL enrichment medium [53]. CuSO_4_ was added at a concentration of 400 mg/L [54] and lincomycin was added at a concentration of 100 mg/L [50] to build an artificial microcosms model with heavy metals and antibiotics. To prevent the photodegradation of antibiotics, enrichment cultures under drug exposure were incubated in the dark at 28 °C for 255 days in individually sealed glass vials. Samples from enrichment culture were obtained for the isolation of bacteria.

### 3.2. Bacterial Isolation and Identification

The samples which were enriched for 255 days were diluted serially with saline and the suspensions were applied to MA which contained different amounts of CuSO_4_ (400 mg/L) and lincomycin (100 mg/L). After incubation at 28 °C for 7 days, different single colonies on the plate were picked, isolated, and purified according to the color, size, and morphology. 

The 16S rRNA gene sequences of the isolates were amplified by polymerase chain reaction (PCR) with two universal primers: 27F and 1492R:27F (59-AGAGTTTGATCMTGG-CTCAG-39) and 1492R (59-TACGGYTACCTTGTTACGA-C-39) [55]. Sequencing of the amplified 16S rRNA gene was conducted by Tsingke Biotechnology Co., Ltd. (Qingdao, China). The 16S rRNA gene sequences were compared by using the EzBioCloud database (http://www.ezbiocloud.net, accessed on 25 October 2022) [56]. The strains were stored in sterile 1% (*w*/*v*) saline supplemented with 15% (*v*/*v*) glycerol at −80 °C.

### 3.3. Bacterial Resistance Assay against Antibiotics and Heavy Metals

Antibiotic and heavy metal resistance of isolates were further studied to screen great multidrug-resistant bacteria. CuSO_4_ (500 mg/L) and lincomycin (300 mg/L) were added in MA, which were autoclaved and filtered for sterilization in turn. A single colony was picked and streaked on selection medium. After incubation at 30 °C for 48 h, the most resistant strain was screened by observing the growth status of bacteria on the culture medium [57,58].

### 3.4. Characterization of a Novel Resistant Strain

#### 3.4.1. Morphological, Physiological and Biochemical Characteristics

According to the Manual for the Systematic Identification of General Bacteria [59], bacterial physiological and biochemical identification was carried out for the most resistant strain ZC255. It includes a test of the hydrolysis of Tweens (20, 40, 60, and 80, 1%, *v*/*v*) and caseins (1%, *w*/*v*), starch (0.2%, *w*/*v*), CM-cellulose (0.5%, *w*/*v*), and alginate (2%, *w*/*v*), and a reduction of nitrate. Other biochemical analyses were performed applying the BIOLOG GEN III MicroPlates, API 50CH, API 20E, and API ZYM (BioMérieux China Ltd., Shanghai, China). All reagent strip tests were performed according to the instructions except that the NaCl concentration was adjusted to the optimum.

Growth at different temperatures (4, 15, 20, 28, 30, 35, 37, 40, 42, and 45 °C) was tested for approximately 7 days on MA medium (growth was recorded every 4 h). Salt tolerance was determined using modified MA (prepared according to the MA formula, but without NaCl) with various concentrations of NaCl (0–20% at 0.5% intervals, *w*/*v*). Growth tests to determine pH range (5.5–9.5 at 0.5 pH unit intervals) were performed in Marine broth 2216 (MB; Becton-Dickinson, Franklin Lakes, NJ, USA) at 30 °C for 2 days on the rotary shaker (ZWY-200D, Zhicheng Analytical Instrument Manufacturing Co., Ltd., Shanghai, China). The pH of the medium was adjusted using a 20 mM concentration of the commercial additional buffer: MES (pH 5.5 and 6.0), PIPES (pH 6.5 and 7.0), HEPES (pH 7.5 and 8.0), Tricine (pH 8.5), and CAPSO (pH 9.0 and 9.5).

#### 3.4.2. Phylogenetic Analysis 

The 16S rRNA gene of strain ZC255 was amplified by PCR with two bacterial universal primers (27F and 1492R) and the purified gene product was cloned using the previously described method to obtain an almost complete 16S rRNA gene sequence. The almost complete 16S rRNA gene of strain ZC255 was purified and sequenced by Tsingke Biotechnology Co., Ltd. (Qingdao, China). The 16S rRNA gene similarities between strain ZC255 and closely related species were calculated using the Biotechnology Information (NCBI) databases (https://www.ncbi.nlm.nih.gov, accessed on 7 June 2023) and EzBioCloud database (http://www.ezbiocloud.net, accessed on 7 June 2023).The 16S rRNA gene sequence of strain ZC255 and those of related strains were aligned by MUSCLE service [60] and phylogenetic trees were produced by bootstrap calculation based on 1000 replicates [61] based on neighbor-joining (NJ) algorithms in MEGA 7 software [62].

#### 3.4.3. Biofilm Formation Assay

The biofilm-forming capacity of the isolates was assayed using a 96-microtiter well plate (BKMAM BIOTECHNOLOGY Co., Ltd., Changsha, China) after 24 h of incubation at 30 °C. They were reconstituted in MA with 100 μg/L Cu (II). After discarding the broth in the wells and washing gently, 150 μL of crystal violet solution (0.1% *w*/*v*) was added. The plate was then dried and photographed [63]. 

#### 3.4.4. Quantifying the Resistance to Different Drugs 

The minimum inhibitory concentration (MIC) assay for the isolate was performed using a 96-microtiter well plate (BKMAM BIOTECHNOLOGY Co., Ltd., Changsha, China) spiked with varying concentrations of Cu (II), Pb (II), Ni (II), Mn (II), Zn (II), Cr (III), and Cd (II). For these, the range of concentration used was 100 to 10,000 mg/L. After incubation at 37 °C for 3 days, the optical density values of 600 nm were measured to determine the corresponding MIC [64]. Then, growth at different Cu (II) concentrations (0, 3840, 5120, 6400, 7680, 8192 mg/L) was determined in MB, and the maximum tolerance concentration (MTC) of Cu (II) for strain ZC255 was measured in this way. The OD values of 600 nm at each concentration were determined and growth curves were made [65].

The resistance to the following antimicrobial agents was determined by the disc-diffusion method [66]: Lincomycin, Carbenicillin, Vancomycin, Norfloxacin, Kanamycin, Ofloxacin, Ampicillin, Penicillin, Polymyxin B, Ceftriaxone, Erythromycin, Chloramphenicol, Streptomycin, Clarithromycin, Rifampin, Cefotaxime sodium, Neomycin, Tobramycin, Tetracycline, and Gentamycin. After 24 h of cultivation and growth, single colonies of strain ZC255 were picked and then were suspended in sterilized artificial seawater to match the McFarland 0.5 turbidity standard. MA plates were inoculated with a suspension of the bacterial isolate. Drug-sensitive papers (6.35 mm) were placed on the surface of the agar plates, and the plates were incubated at 37 °C for 48 h to determine whether the strain is resistant to drugs. Then, growth at different lincomycin concentration (0, 200, 400, 600, 850, and 900 mg/L) were further monitored in MB, and the MTC of lincomycin for strain ZC255 was measured in this way. The OD_600_ values at each concentration were determined and growth curves were made.

#### 3.4.5. Bioremoval Experiment

Cu (II) and lincomycin were selected as representatives of heavy metals and antibiotics to test the bioremediation ability of strain ZC255. The batch bioremoval of heavy metal Cu (II) and lincomycin by strain ZC255 was conducted at a low- and high-initial concentration, 1000 mg/L and 3500 mg/L for Cu (II) and 200 mg/L and 400 mg/L for lincomycin, respectively. After treatment at 37 °C for 3 days, supernatant was harvested by centrifugation at 8000 r/min for 5 min. The supernatants were subjected to atomic absorption spectrophotometer (Model: 900H, Perkin Elmer, Waltham, MA, USA) analysis to measure the residual concentration of Cu (II) and subjected to LC-30AD liquid chromatograph (Shimadzu Co., Kyoto, Japan) and API4000 Q-TRAP mass spectrometer (AB SCIEX, San Francisco, CA, USA) analysis to measure the residual concentration of lincomycin [67]. The adsorption rate and absolute adsorption capacity were calculated [68].

Strain’s bioremoval rate (R) and bioremoval capacity (Q) of Cu (II) and lincomycin:

R = (1 − *C_t_*/*C*_0_) × 100%

Q = (*C*_0_ − *C_t_*) × *V*/*M*

where R is the bioremoval rate (%), *C_t_* is the final metal concentration (mg/L), *C*_0_ is the initial metal concentration (mg/L), *V* is the volume of medium (mL), *M* is the biomass of strain ZC255 (g), and Q is the bioremoval capacity of the strain ZC255 (mg/g). All experiments were performed in triplicate and the data were expressed as mean ± standard deviation (SD) to validate data variability and validation data. All data were tested for significance.

## 4. Conclusions

This study demonstrated that after 255 days of enrichment under Cu and lincomycin stress, only the *Bacillaceae* species were isolated. It was obvious that the microflora of marine sediments was greatly affected.

ZC255 was the most resistant strain obtained. The 16S rRNA gene sequence confirmed that it belonged to the genus *Rossellomore*. Strain ZC255 exhibited strong multidrug resistance and bioremoval ability for heavy metals and antibiotics. These suggest that strain ZC255 is a promising alternative in biotechnological applications for the bioremediation of contaminated environments.

## Figures and Tables

**Table 1:** Minimal inhibitory concentrations (MIC) and maximum tolerated concentrations (MTC) of heavy metal ions for the growth of strain ZC255.

Heavy Metals	MIC (mg/L)	MTC (mg/L)
Zn (II)	600	2660
Cu (II)	1600	7680
Ni (II)	400	1210
Cr (III)	320	820
Cd (II)	100	400
Pb (II)	440	1640
Mn (II)	10	20

**Table 2:** Antibiotics sensitive tests of strain ZC255 drug sensitive test.

Antibiotics	Test Result ^a^
Lincomycin	R
Carbenicillin	S
Vancomycin	S
Norfloxacin	R
Kanamycin	R
Ofloxacin	R
Ampicillin	S
Penicillin	S
Polymyxin B	R
Ceftriaxone	R
Erythromycin	R
Chloramphenicol	S
Streptomycin	R
Clarithromycin	S
Rifampin	I
Clarithromycin	I
Neomycin	R
Tobramycin	R
Tetracycline	R
Gentamycin	R

^a^ R denotes resistance, I denotes intermediate, and S denotes susceptible.

**Table 3:** The removal efficiency of Cu (II) by strain ZC255.

Metal Ion	Initial Concentration (mg/L)	Final Concentration (mg/L)	Bioremoval Percentage	Bioremoval Capacity (mg/g Biomass)
Cu (II)	1200	1000	16.67%	350.2 ± 2.6
	3420	3234	5.43%	651.4 ± 1.7

**Table 4:** The removal efficiency of lincomycin by strain ZC255.

Antibiotics	Initial Concentration (mg/L)	Final Concentration (mg/L)	Bioremoval Percentage	Bioremoval Capacity (mg/g Biomass)
Lincomycin	424	385	9.19%	32.5 ± 0.4
	199	187	6.03%	8.1 ± 0.1

## Data Availability

Not applicable.

## Supplementary Materials

The following supporting information can be downloaded at: https://www.mdpi.com/article/10.3390/antibiotics12091379/s1, Figure S1: Colony morphology of strain ZC255; Figure S2: Growth curve of strain ZC255 at different pH value; Figure S3: Antibiotics sensitive tests of strain ZC255; Table S1: Different characteristics of strain ZC255; Table S2: Reference standard of Susceptibility testing.
