# Supplementary material for: Antibiotic and Heavy Metal Co-Resistant Strain Isolated from Enrichment Culture of Marine Sediments, with Potential for Environmental Bioremediation Applications
Source: Antibiotics (Basel). 2023 Aug 29;12(9):1379. doi: 10.3390/antibiotics12091379 (PMC10526090; doi:10.3390/antibiotics12091379)
Supplement: Supplementary file 1 [file antibiotics-12-01379-s001.zip › antibiotics-2552969-supplementary.pdf]

# Supplementary material

## Antibiotic and heavy metal co-resistant strain isolated from enrichment culture of marine sediments, with potential for environmental bioremediation applications

**Han-Sheng Zhu[1], Xiao Liang[1], Jun-Cheng Liu[2], Han-Yang Zhong[1], Yuan-Hang Yang[1], Wen-Peng Guan[1], Zong-Jun Du[1,3]*, Meng-Qi Ye[1,3,4]***

[1]Marine College, Shandong University, Weihai, Shandong, 264209,PR China;

[2]SDU-ANU Joint Science College, Shandong University, Weihai, Shandong, 264209, PR China

[3]Weihai Research Institute of Industrial Technology of Shandong University, Weihai, Shandong, 264209, PR China; Shenzhen Research Institute of Shandong University, Shenzhen, Guangdong, 518057, PR China;

[4]Shenzhen Research Institute of Shandong University, Shenzhen, Guangdong, 518057, PR China;

*Correspondence: yemengqi@126.com（M.-Q.-Y）

duzongjun@sdu.edu.cn（Z.-J.-D.）

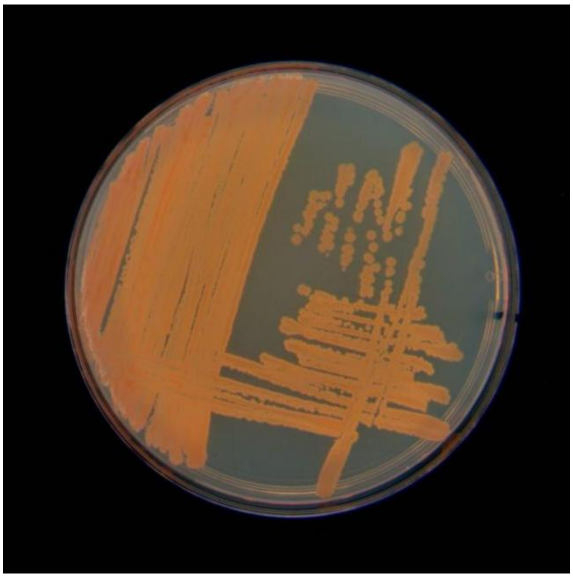

**Figure S1** Colony morphology of strain ZC255

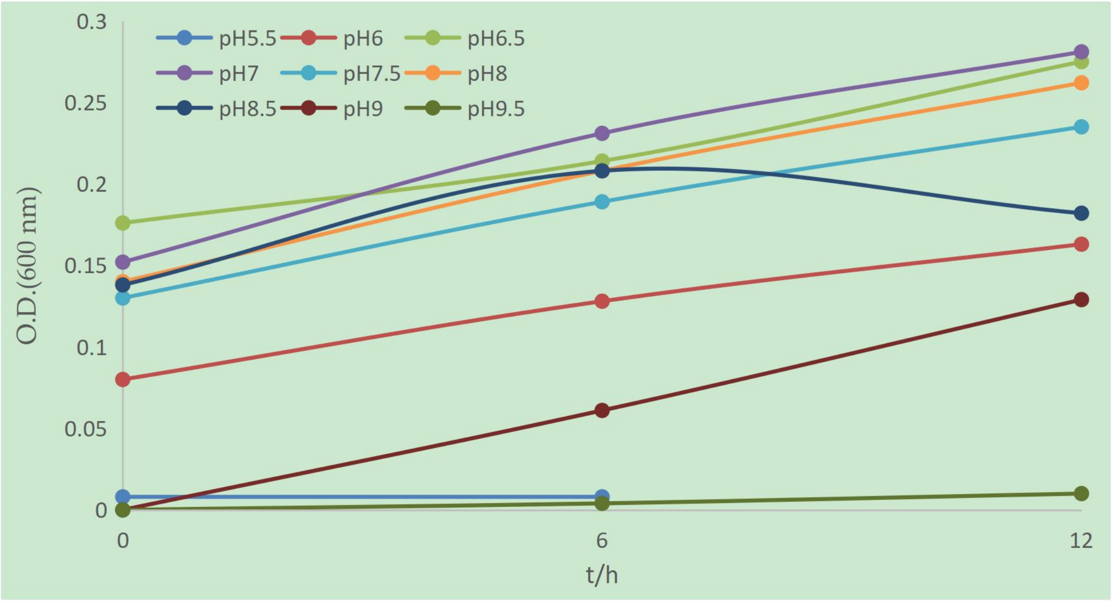


**Figure S2** Growth curve of strain ZC255 at different pH value

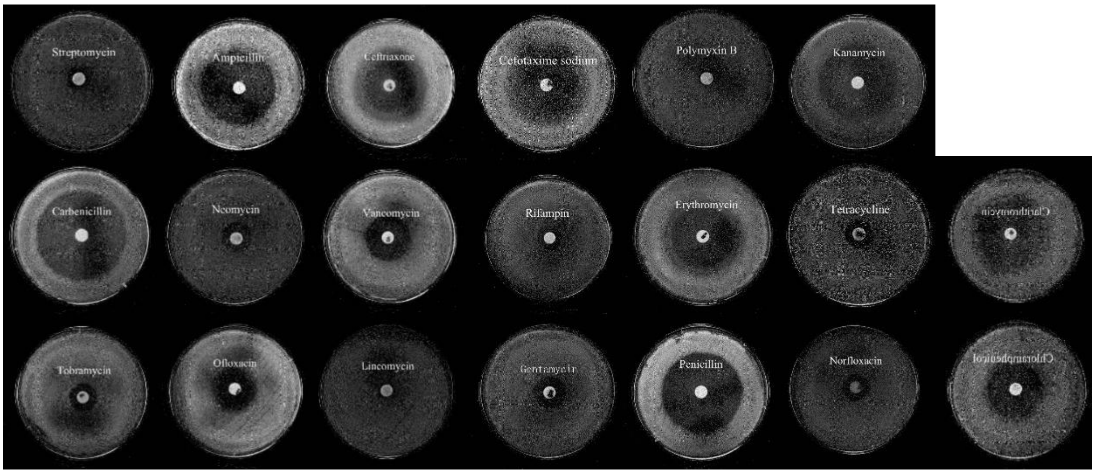


**Figure S3** Antibiotics sensitive tests of strain ZC255

**Table S1** Different characteristics of strain ZC255

| Strain | | ZC255 |
|---|---|---|
| | API 20E | |
| ONPG | *β*-Galactosidase | - |
| ADH | Arginine dihydrolase | - |
| LDC | Lysine decarboxylase | - |
| ODC | Ornithine decarboxylation | - |
| CIT | Citrate utilization | + |
| H2S | $H_2S$ production | - |
| URE | urease | + |
| TDA | Tryptophan deaminase | + |
| IND | Indole production | - |
| VP | Voges-Proskauer reaction | - |
| GEL | Gelatin | + |
| GLU | glucose | - |
| MAN | mannitol | - |
| INO | inositol | - |
| SOR | sorbitol | - |
| RHA | rhamnol | - |
| SAC | sucrose | - |
| MEL | melibiose | - |
| AMY | amygdalin | - |

| | | |
|---|---|---|
| ARA | arabinose | - |
| | Biolog GEN III MicroPlate | |
| 1A | negative-control | w |
| 2A | dextrin | w |
| 3A | D-maltose | + |
| 4A | D-trehalose | + |
| 5A | D-cellobiose | + |
| 6A | gentiobioswe | - |
| 7A | sucrose | + |
| 8A | D-turanose | + |
| 9A | stachyose | + |
| 10A | positive control | + |
| 11A | pH 6 | w |
| 12A | pH 5 | - |
| 1B | D-raffinose | w |
| 2B | D-lactose | w |
| 3B | D-melibiose | w |
| 4B | *β*-methyl-D-glucoside | w |
| 5B | D-salicin | - |
| 6B | N-acetyl-D-glucosamine | + |
| 7B | N-acetyl-*β*-D-mannosamine | + |
| 8B | N-acetyl-D-galactosamine | + |

| | | |
|---|---|---|
| 9B | N-acetyl neuraminic acid | + |
| 10B | 1% NaCl | + |
| 11B | 4% NaCl | w |
| 12B | 8% NaCl | - |
| 1C | $\alpha$-D-glucose | - |
| 2C | D-mannose | + |
| 3C | D-fructose | + |
| 4C | D-galactose | + |
| 5C | 3-methyl glucose | + |
| 6C | D-fucose | + |
| 7C | L-fucose | + |
| 8C | L-rhamnose | + |
| 9C | inosine | + |
| 10C | 1% sodium lactate | - |
| 11C | fusidic acid | - |
| 12C | D-serine | - |
| 1D | D-sorbitol | + |
| 2D | D-mannitol | w |
| 3D | D-arabitol | w |
| 4D | myo-inositol | + |
| 5D | glycerol | - |
| 6D | D-glucose-6-$PO_4$ | - |

| | | |
|---|---|---|
| 7D | D-fructose-6-$PO_4$ | - |
| 8D | D-aspartic acid | w |
| 9D | D-serine | - |
| 10D | troleandomycin | - |
| 11D | rifamycin SV | - |
| 12D | minocycline | - |
| 1E | gelatin | - |
| 2E | glycyl- L-proline | - |
| 3E | L-alanine | w |
| 4E | L-arginine | w |
| 5E | L-aspartic acid | w |
| 6E | L-glutamic acid | w |
| 7E | L-histidine | - |
| 8E | L-pyroglutamic acid | w |
| 9E | L-serine | w |
| 10E | lincomycin | - |
| 11E | guanidine HCl | - |
| 12E | niaproof 4 | - |
| 1F | pectin | + |
| 2F | D-galacturonic acid | w |
| 3F | L-galactonic acid lactone | - |
| 4F | D-gluconic acid | + |

| | | |
|---|---|---|
| 5F | D-glucuronic acid | - |
| 6F | glucuronamide | - |
| 7F | mucic acid | w |
| 8F | quinic acid | w |
| 9F | D-saccharic acid | w |
| 10F | vancomycin | - |
| 11F | tetrazolium violet | - |
| 12F | tetrazolium blue | - |
| 1G | $\rho$-hydroxy-phenylacectic acid | - |
| 2G | methyl pyruvate | - |
| 3G | D-lactic acid methyl ester | w |
| 4G | L-lactic acid | w |
| 5G | citric acid | - |
| 6G | $\alpha$-keto-glutaric acid | w |
| 7G | D-malic acid | w |
| 8G | L-malic acid | w |
| 9G | bromo-succinic acid | - |
| 10G | nalidixic acid | - |
| 11G | lithium chloride | - |
| 12G | potassium tellurite | - |
| 1H | tween-40 | - |
| 2H | $\gamma$-amino-butyric acid | - |

| | | |
|---|---|---|
| 3H | $\alpha$-hydroxy-butyric acid | - |
| 4H | $\beta$-hydroxy-D, L-butyric acid | w |
| 5H | $\alpha$-keto-butyric acid | - |
| 6H | acetoacetic acid | + |
| 7H | propionic acid | - |
| 8H | acetic acid | + |
| 9H | formic acid | - |
| 10H | aztreonam | - |
| 11H | sodium butyrate | - |
| 12H | Sodium bromate | - |
| | API 50 CH | |
| 1 | GLY | + |
| 2 | ERY | - |
| 3 | DARA | - |
| 4 | LARA | - |
| 5 | RIB | + |
| 6 | DXYL | - |
| 7 | LXYL | - |
| 8 | ADO | - |
| 9 | MDX | - |
| 10 | GAL | + |
| 11 | GLU | + |

| | | |
|---|---|---|
| 12 | FRU | + |
| 13 | MNE | + |
| 14 | SBE | - |
| 15 | RHA | - |
| 16 | DUL | - |
| 17 | INO | - |
| 18 | MAN | - |
| 19 | SOB | - |
| 20 | MDM | - |
| 21 | MDG | + |
| 22 | NAG | + |
| 23 | AMY | + |
| 24 | ARB | + |
| 25 | ESC | + |
| 26 | SAL | + |
| 27 | CEL | + |
| 28 | MAL | + |
| 29 | LAC | + |
| 30 | MEL | + |
| 31 | SAC | + |
| 32 | TRE | + |
| 33 | INU | - |

| | | |
|---|---|---|
| 34 | MLZ | + |
| 35 | RAF | + |
| 36 | AMD | + |
| 37 | GLYG | + |
| 38 | XLT | - |
| 39 | GEN | + |
| 40 | TUR | + |
| 41 | LYX | - |
| 42 | TAG | + |
| 43 | DFUC | - |
| 44 | LFUC | - |
| 45 | DARL | - |
| 46 | LARL | - |
| 47 | GNT | - |
| 48 | 2KG | - |
| 49 | 5KG | + |
| | API ZYM | |
| 1 | 2-Naphthyl phosphate | - |
| 2 | 2-Naphthyl butyrate | - |
| 3 | 2-Naphthyl octanoate | + |
| 4 | 2-Naphthyl tetradecanoate | + |
| 5 | L-Leucinyl-2-naphthylamine | - |

| | | |
|---|---|---|
| 6 | L-Valinyl-2-naphthylamine | - |
| 7 | L-Cystinyl-2-naphthylamine | - |
| 8 | N-Benzoyl-DL-argininyl-2-naphthylamine | - |
| 9 | N-Shodanoyl-phenylalanine2-naphthylamine | - |
| 10 | N-Naphthyl-phosphate | + |
| 11 | Naphthol-AS-BI-phosphate | - |
| 12 | 6-Bromo-2-naphthyl-alpha D-galactopyranoside | - |
| 13 | 2-Naphthyl-β D-galactopyranoside | - |
| 14 | Naphthol-AS-β-mono-D-glucuronide | - |
| 15 | 2-Naphthyl-alpha D-glucopyranoside | - |
| 16 | 6-Bromo-2-naphthyl-β D-glucopyranoside | - |
| 17 | 1-Naphthyl-N-acetyl-β D-glucosamine | - |
| 18 | 6-Bromo-2-naphthyl-alpha D-mannopyranoside | - |
| 19 | 2-Naphthyl-alpha L-fructopyranoside | - |

Table S2 Reference standard of Susceptibility testing

| Antibiotics | Drug content | Bacteriostatic circle diameter (mm) | | |
|---|---|---|---|---|
| | (ug/disk) | Resistance | Intermedi | Susceptibl |
| Lincomycin | 2 | 12 | 13-15 | 15 |
| Carbenicillin | 100 | 19 | 20-22 | 23 |
| Vancomycin | 30 | 14 | 15-16 | 17 |
| Norfloxacin | 30 | 12 | 13-16 | 17 |
| Kanamycin | 30 | 13 | 14-17 | 18 |
| Ofloxacin | 5 | 12 | 13-17 | 18 |
| Ampicillin | 10 | 13 | 14-16 | 17 |
| Penicillin | 10 | 14 | | 15 |
| Polymyxin B | 300 | 8 | 10 | 12 |
| Ceftriaxone | 30 | 13 | 14-20 | 21 |
| Erythromycin | 15 | 13 | 14-22 | ≥23 |
| Chloramphenicol | 30 | 12 | 13-17 | 18 |
| Streptomycin | 10 | 11 | 12-14 | 15 |
| Clarithromycin | 10 | 13 | 14-17 | 18 |
| Rifampin | 5 | 16 | 17-19 | 20 |
| Cefotaxime sodium | 30 | 14 | 15-25 | 26 |
| Neomycin | 30 | 13 | 14-18 | 19 |
| Tobramycin | 10 | 12 | 13-14 | 15 |

| | | | | |
|---|---|---|---|---|
| Tetracycline | 30 | 14 | 15-18 | 19 |
| Gentamycin | 10 | 12 | 13-14 | 15 |
